# The trans-european catchment area of common noctule bats killed by wind turbines in France

**DOI:** 10.1038/s41598-025-85636-5

**Published:** 2025-01-09

**Authors:** Maela Merlet, David X. Soto, Laurent Arthur, Christian C. Voigt

**Affiliations:** 1 Leibniz Institute for Zoo and Wildlife Research (IZW), Alfred-Kowalke-Str. 17, 10315 Berlin, Germany; 2 Chauve-Qui-Peut, 22 rue Ranchot, L’observatoire, Bourges, 18000 France; 3 Universität Potsdam, Am Mühlenberg 3, 14476 Potsdam, Germany

**Keywords:** Conservation, Bat migration, Deuterium, IsoriX, Stable isotopes, Ecology, Zoology, Ecology

## Abstract

**Wind turbines used to combat climate change pose a green-green dilemma when endangered and protected wildlife species are killed by collisions with rotating blades. Here**,** we investigated the geographic origin of bats killed by wind turbines along an east-west transect in France to determine the spatial extent of this conflict in Western Europe. We analysed stable hydrogen isotopes in the fur keratin of 60 common noctule bats (*Nyctalus noctula*) killed by wind turbines during summer migration in four regions of France to predict their geographic origin using models based on precipitation isoscapes. We first separated migratory from regional individuals based on fur isotope ratios of local bats. Across all regions**,** 71.7% of common noctules killed by turbines were of regional and 28.3% of distant origin**,** the latter being predominantly females from northeastern Europe. We observed a higher proportion of migratory individuals from western sites compared to eastern sites. Our study suggests that wind-turbine-related losses of common noctule bats may impact distant breeding populations across whole Europe**,** confirming that migratory bats are highly vulnerable to wind turbines and that effective conservation measures**,** such as temporary curtailment of turbine operation**,** should be mandatory to protect them from colliding with the rotating blades of wind turbines.**

Our planet is facing several major environmental crises that threaten ecosystems and jeopardise the survival of humanity^1,2^. Solving these environmental crises should therefore be of primary concern to society^3^. The biodiversity crisis, commonly known as the sixth mass extinction event^4^, is mainly caused by land-use changes and intensification^5,6^, processes that are widely underestimated in their impact on biodiversity^7^. While more and more land is being converted or used more intensively for human needs, ecosystems are also increasingly suffering from global warming and unpredictable weather extremes^8^. Measures intended to solve one of these specific environmental crises may conflict with the solution to the other environmental crisis, which is commonly referred to as a green-green dilemma^9^. One such dilemma lies in the fact that many threatened and legally protected wildlife species are killed by wind turbines^10^. Indeed, an increasing number of studies suggests that wind energy production is leading to habitat losses and degradation and to casualties of vulnerable wildlife worldwide^1–13^.

Over the past two decades high fatality rates have been documented for bats and birds at wind turbines on all continents where wind turbines are operating^9,10,14^. In fact, it has been suggested that turbine-induced casualties may represent the most likely cause for multiple mortality events in bats^15^. In line with this notion, past studies have estimated that each year millions of bats may get killed at wind turbine facilities of the northern hemisphere^9^. This massive loss of individuals may translate into population declines of high collision risk species because of the high vulnerability of female and juvenile bats^16^and the overall low reproductive rate of bats, as illustrated by studies from North America and Europe^17–19^. This problem could be further exacerbated if wind energy production encroaches on ecologically sensitive areas such as forests^20^. Forests are an important habitat for bats worldwide^21,22^, so it is foreseeable that wind energy expansion at forested sites could lead to a higher number of casualties of bats and greater habitat loss, both through clearing and the avoidance behaviour of bats towards operating wind turbines^23–26^. Overall, the conflict scenario between wind energy production and bat conservation is unfortunate given the availability of effective avoidance and mitigation measures. For example, guidelines recommend that wind turbines should be placed at a great distance from ecologically sensitive areas^20^. In addition, wind turbines should be temporarily shut down during periods of high bat activity, a mitigation measure called curtailment^27^. Before the critical wind speed for power generation of wind turbines is reached, the blades should be turned at an angle to the incoming wind that reduces the airflow that causes the blades to rotate. This process, called blade feathering, ensures that bats do not collide with the moving blades as the rotation is kept minimal^20^. However, these measures are not systematically applied in any of the areas where wind energy production is expanding^9,28,29^.

The noctule bat (*Nyctalus noctula*) is one of the most common bat species found dead under wind turbines in Central Europe^28,30,31^. This species is an aerial insectivorous bat that hunts above arable land, forests and urban areas^32–39^. It is also known for its migratory behaviour. In northeastern Europe, where summer populations consist mainly of reproducing females, all individuals migrate to Central and Western Europe in autumn^40,41^. Whereas in Central Europe, the common noctule forms partially migratory populations, i.e. in late summer, colonies may consist of migratory individuals originating from northeastern populations, resident individuals that remain year-round in the region and others that migrate to Western Europe in autumn^42^. During migration, common noctule bats cover several hundred kilometres between their summer and wintering habitat^40,41^. Some banded individuals have also been found travelling distances of up to 1,500 km in one direction^40,41^. As a migratory bat species, common noctules are particularly affected by the expansion of wind energy production in Europe^43–45^, because their flight altitude at 30 to 200 m above ground overlaps largely with the rotor-swept area of turbines^34,35,46,47^. Current monitoring schemes and analyses report a 53% decrease in population size between 2006 and 2023 in France, and also declines in central Europe for this species^19,48,49^. Also, previous geographic assignments indicated that a relatively large proportion of common noctule bats killed by wind turbines in Central Europa originates from the summer range in northeastern Europe^43,50^. Due to their cryptic roosting behaviour in tree hollows^33^, population monitoring is highly difficult in their summer range. It is therefore important to determine the proportion of migrating individuals in those regions where wind energy production is expanding to assess the potential impact of casualties by wind turbine for remote and local populations.

Here, we ask what the relative proportion of long-distance migratory bats is among bats killed by wind turbines in four regions of France, stretching from west to east from Bretagne/Pays-de-la-Loire to Bourgogne-Franche-Comté/Grand-Est, covering a distance of about 500 km in west-east direction across France. We hypothesised that wind turbines in France kill not only local (hereafter referred to as regional) common noctule bats, but also long-distance migrants, thus affecting populations at a large spatial scale in central and north-eastern Europe. Given that France is in the western geographic range of common noctule bats in Europe^33^, we predicted that the relative proportion of long-distance migratory common noctule bats killed by wind turbines in France would be similar to or even higher than observed for common noctule bats killed by wind turbines in Germany (28% of all carcasses studied^50^). Secondly, we predicted that the relative proportion of long-distance migratory common noctules is higher at the western than at the eastern study sites in France, because the western study sites are at the border of the species’ geographic range, leading potentially to a higher proportion of migratory to regional bats. We used stable isotopes to shed light on the geographic origin of bats, because stable hydrogen isotope ratios, depicted in the delta notation (δ^2^H) as per mille deviation from an international standard, in fur keratin inform about the geographic area of the summer range of bats^51^. Fur is a suitable matrix for this approach, as stable isotopes are conserved in the inert keratin after growth^52^. Furthermore, we use stable carbon and nitrogen isotope ratios (δ^13^C and δ^15^N values, respectively) to elucidate the extent of foraging on limnic or terrestrial insects in common noctule bats across Europe^53,54^. Fur keratin enriched in ^15^N and depleted in ^13^C, is indicative of foraging on insects with limnic larval stages^54^.

Our study is particularly important for stakeholders and political decision makers since common noctule bats are not only protected by French legislation (Loi de protection de la nature, § 76–629, 1976), but also by international legislation (EU habitat directive, 92/43/EWG). Further to that, European migratory bats are covered by the Convention on Migratory Species of wild animals (CMS convention, UNEP/EUROBATS agreement, signed Bonn 1979, and London 1991). Accordingly, the protection of this and other migratory bats should be in the core interest of national and international conservation efforts of authorities and governments.

## Results

### Variations of δ^2^H, δ^13^C, and δ^15^N values in fur keratin

In total, our study is based on 60 carcasses from four different sites within the geographic range of common noctule bats in France (Table 1). Stable isotope values in fur keratin of common noctules ranged from − 74.3‰ to −25.0‰ for δ^2^H, from − 27.4‰ to −21.4‰ for δ^13^C, and 6.8‰ to 13.6‰ for δ^15^N values. δ^2^H and δ^15^N values were negatively correlated (Spearman’s r_57_ = −0.36, *p*= 0.005), while δ^2^H and δ^13^C values showed a weak positive correlation (Spearman’s r_57_ = 0.30, *p*= 0.020). No correlation was found between δ^13^C and δ^15^N values (Pearson’s r_57_ = −0.17, *p* = 0.205).

**Table 1:** Number of bats assigned to the clusters of origin in each sampling group.

	Cluster 1	Cluster 2	Cluster 3	Cluster 4	Cluster 5
G1	0	2	0	2	1
G2	2	4	1	2	1
G3	9	14	10	6	3
G4	2	1	0	0	0
**Total**	**13 (22%)**	**21 (35%)**	**11 (18%)**	**10 (17%)**	**5 (8%)**

Compared to the fur keratin of regional common noctule bats, the fur keratin of long-distance migratory bats was depleted in ^2^H, on average, by 23.7‰ and by 0.7‰ in ^13^C (t_57_= −2.68, *p*= 0.009), while it was enriched in ^15^N, on average, by 1.3‰ (t_26.3_ = 3.57, *p*< 0.001) in relation to the corresponding lighter isotopes. The isotopic composition of fur keratin did not differ between sexes (δ^2^H: U = 149.5, *p*= 0.284; δ^13^C: t_39_ = −1.20, *p*= 0.239; and δ^15^N: t_39_ = 1.31, *p*= 0.199) or vary over time within a year (δ^2^H: F_1,58_ = 0.004, *P*= 0.95; δ^13^C: F_1,57_ = 0.01, *P*= 0.92; and δ^15^N: F_1,57_ = 0.02, *P* = 0.89).

**Fig. 1:**
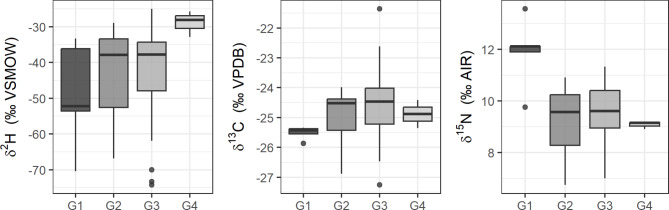
Differences in the δ^2^H, δ^13^C and δ^15^N values in the fur keratin of noctule bats from the four sampling groups (G1 Bretagne/Pays-de-la-Loire; G2 and G3: Centre-Val de Loire north and south; G4 Bourgogne-Franche-Comté/Grand-Est).

We found differences in δ^15^N values between sampling groups (F_3,55_ = 7.14, *p*< 0.001) with bats collected in Western France (G1) presenting higher δ^15^N values than all other groups (Fig. 1). We observed no significant differences in δ^2^H and δ^13^C values between sampling groups (H_3_ = 6.91, *p* = 0.075; and F_3,55_ = 1.04, *p* = 0.383, respectively).

### Relative proportion of regional and long-distance migratory bats

We categorized 71.7% (*n* = 43) of the bats collected in wind farms as of regional origin and 28.3% (*n* = 17) as of long-distance migratory origin. The sex ratio in our sample collection and among migratory individuals was female-biased (Χ² = 4.1, *p* = 0.042 and Χ² = 4.5, *p* = 0.035, respectively). The proportion of females was greater among migratory individuals compared to the total sample (82% and 67%, respectively). Among regional individuals, the female to male ratio was more balanced (60% females, 40% males, Χ² = 1.2, *p*= 0.273). Bats collected in Western France (G1) presented the highest proportion of migrants (60%) while none of the bats from the easternmost sampling group (G4) were categorized as migratory, owing to their high δ^2^H values. Bats from sampling groups in central France, G2 and G3, had intermediate proportions of migratory bats (30% and 26%, respectively).

## Isotope-based geographic assignments

**Fig. 2:**
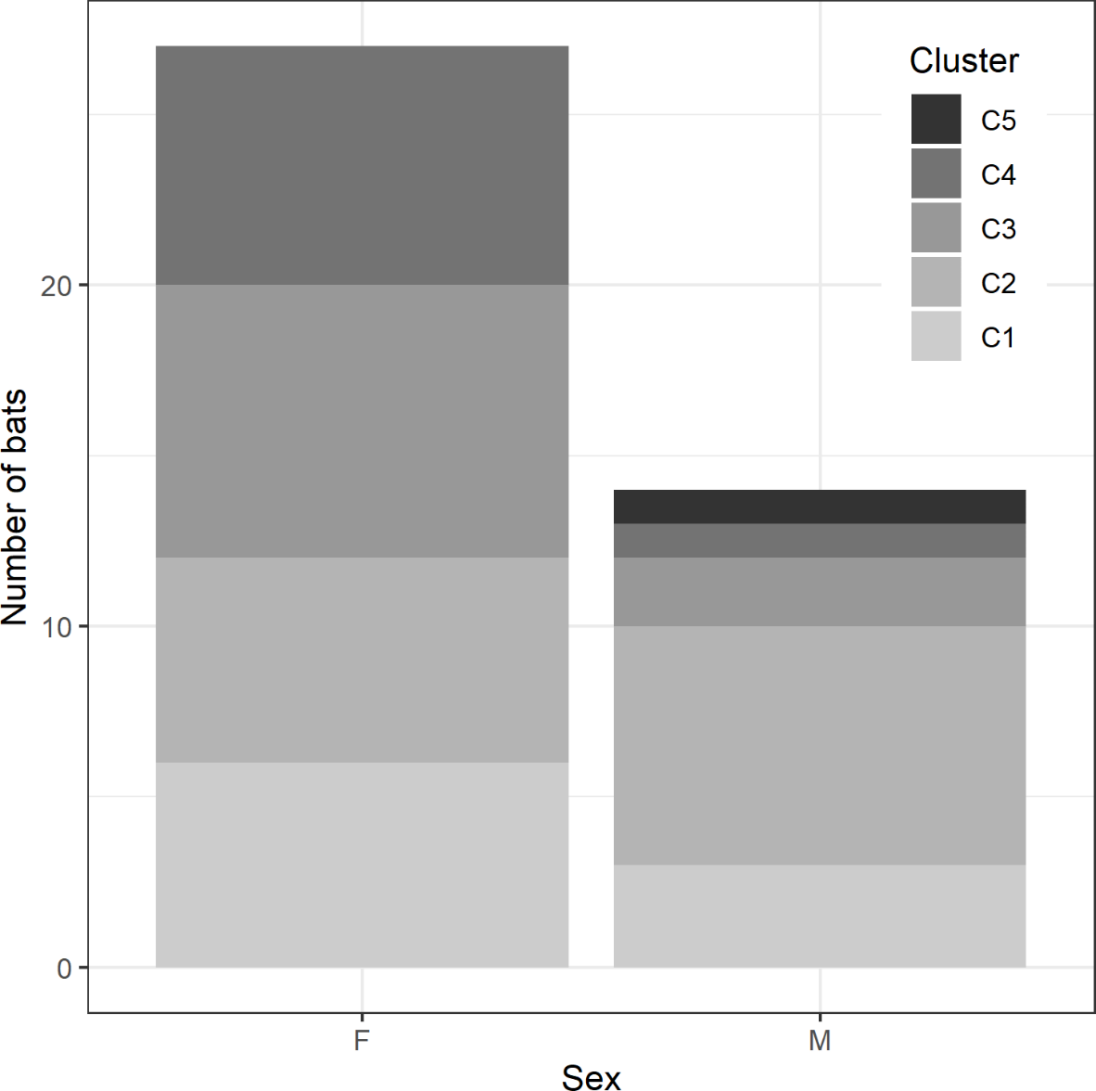
Number of female (F) and male (M) common noctule bats originating from five isotopic clusters.

**Fig. 3:**
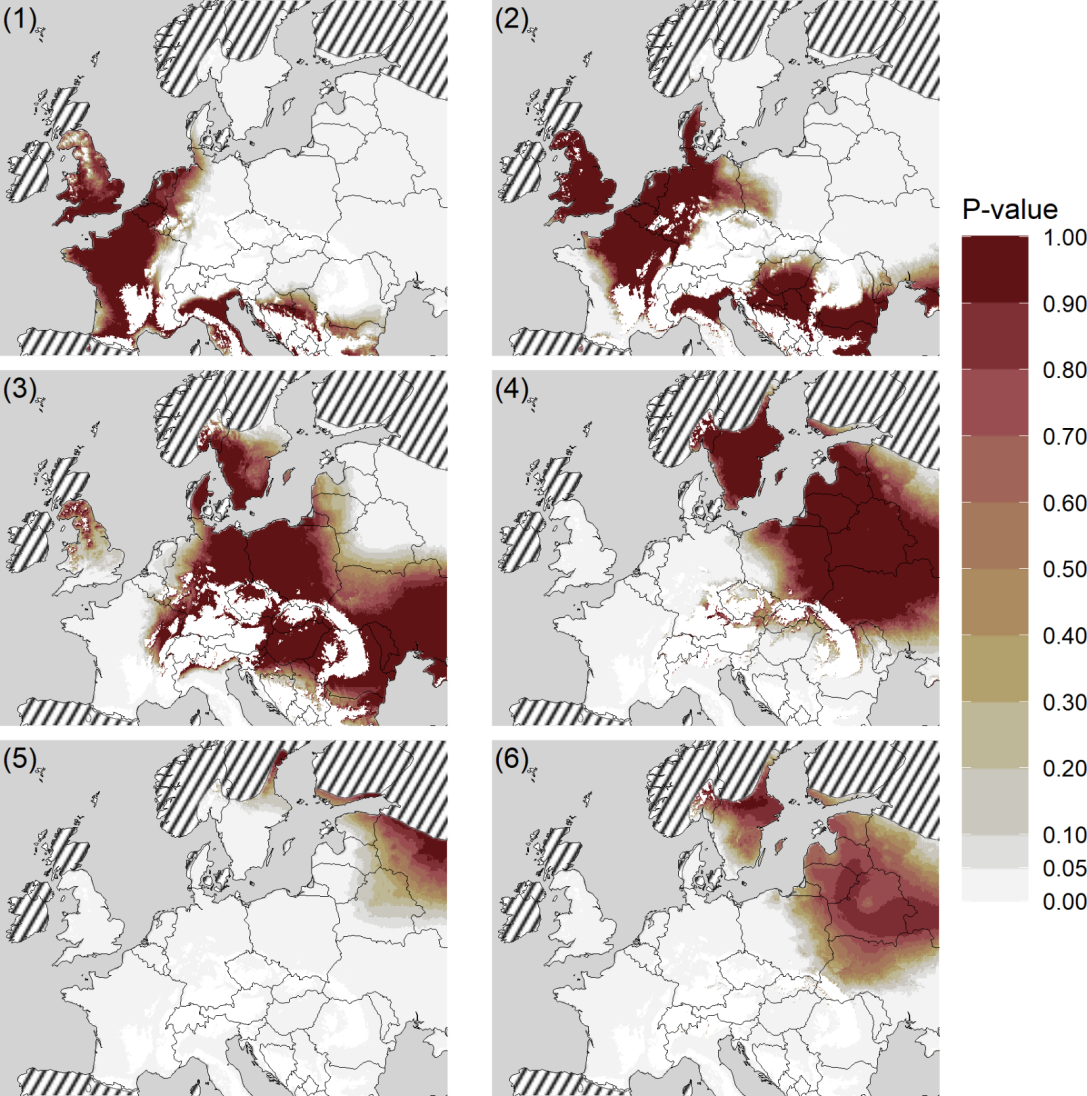
Inferred geographic origin of *Nyctalus noctula* killed in wind farms in France. Areas of likely summer origin of bats from each cluster (1–5) and of all long-distance migratory bats (6). Mountains with elevations above 400 m were excluded from the assignment maps and appear in white. The striped layer represent the areas outside of the species distribution range. Indicated P-values relate to the Probability of occurring at locations, ranging from 0 to 100%, or low (white shaded) to high probability (red) of geographic assignment, respectively.

Common noctule bats were separated into five probable areas of origin using k-means clustering analysis: cluster 1 (centroid δ²H = −29.2‰, *n* = 13), cluster 2 (centroid δ²H = −35.8‰, *n* = 21), cluster 3 (centroid δ²H = −45.4‰, *n* = 11), cluster 4 (centroid δ²H = −55.7‰, *n* = 10), and cluster 5 (centroid δ²H = −71‰, *n* = 5, Table 1). The clusters explained 96.7% of the variation in δ²H values. Males originated predominantly from cluster 2, while females were more equally distributed between clusters 1 to 4 (Fig. 2). Among the common noctule bats included in this study, 57% most likely originated from Southern and Western Europe (clusters 1 and 2) and 43% originated from Central, Northern and Eastern Europe (clusters 3–5) (Table 1; Fig. 3).

## Discussion

Our study aimed to contribute to a better understanding of how wind turbines in Western Europe may impact distant population of migratory species, using common noctule bats as an example for a migratory bat species. Our study shows that about 28% of all common noctules (*n*= 60) included in our study originated from distant locations in northeastern Europe, which is consistent with data from common noctule carcasses found at wind turbines in Germany^50^. Our geographic assignments suggest a likely origin of long-distance migrants from Romania, Poland, Belarus, Russia, the Baltic countries and Fennoscandia, similar to findings from past studies on the geographic origin of long-distance migrating noctule bats in Europe^45,50,55^. Our study therefore goes beyond previous findings by suggesting that the migratory movements of common noctule bats killed at wind turbines cover the entire European continent in a west-east direction. We also observed an increase in the relative proportion of long-distance migrants at collection sites in western France, in line with our prediction. Our observation of a relatively large portion of long-distance migratory common noctule bats among the studied carcasses highlights that current permitting procedures in France do not adequately protect migratory bats by enforcing mitigation measures such as curtailment. This is surprising given the fact that this species is covered by national and international legislation and conventions, including the CMS Convention under UNEP/EUROBATS Agreement (signed Bonn 1979, and London 1991). We therefore agree with the recent conclusions that current European wind energy practices are failing to effectively protect endangered and legally protected bat species^29^. Our study also corroborates previous work on other migratory bat species, which showed a higher proportion of female than male bats among carcasses found at wind turbines in Europe^16^. The relatively high proportion of females among long-distance migrants highlights the fact that the north-eastern populations of this species consist predominantly of reproducing females. We therefore expect that the potential impact of wind turbine fatalities in France could lead to significant declines in the affected populations, as juvenile production is driven by the number of females in the colonies. This could exacerbate the predicted population declines for migratory bats^17,18^, particularly for populations at the northeastern edge of the species’ range.

We also observed high δ^15^N and low δ^13^C values in long-distance migrating noctule bats, suggesting that these bats feed mainly on insects with limnic larval stages in their summer range of northeastern Europe^53,54^, e.g., insects emerging from lakes and peat swamps of the boreal zone. In contrast, stable isotope ratios indicated a higher proportion of terrestrial insects in the diet of regional conspecifics^53,54^. Feeding on insects with limnic larval stages may provide long-distance migrating bats with a relatively high input of polyunsaturated fatty acids, such as linoleic acid, which are useful for torpor and endurance exercise^56,57^, and which are rare in the mammalian body in general, and in that of common noctule bats specifically^58^.

**Fig. 4:**
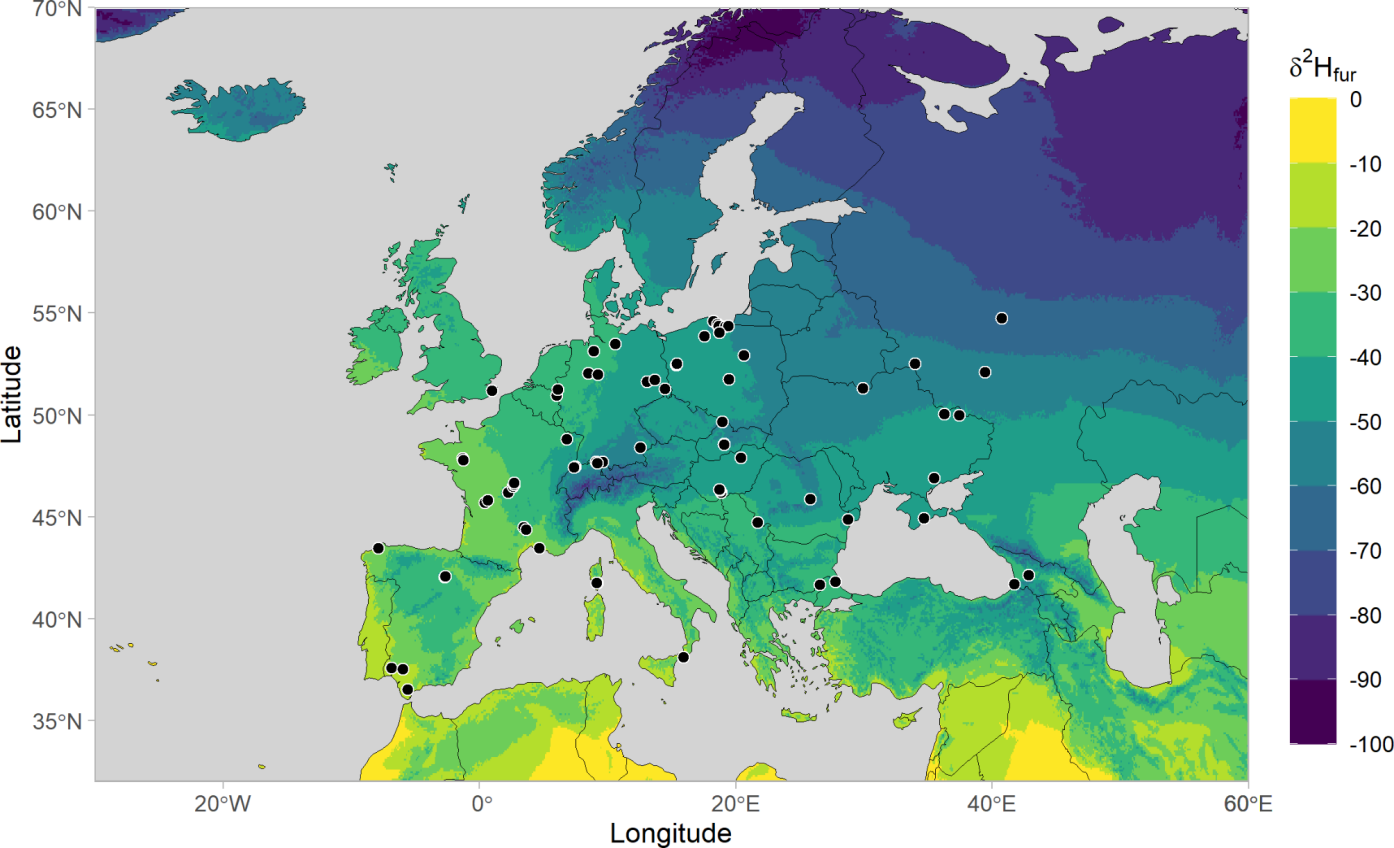
Bat fur δ^2^H model isoscape for Europe. The prediction raster was based on the amount-weighted mean annual hydrogen isoscape in precipitation modelled in this study using IsoriX and the derived transfer function from water to bat tissue H. Black dots indicate the sampling locations of the 335 known-origin European bats included in the transfer function.

In our stable isotope approach, we used a combination of elements to offer insights into the ecology and geographic origin of animals. The value of this approach for bat species has been confirmed in previous work^42,43,45,50,59–61^. Although the isotopic approach is robust for geographic assignment of bats^51^, it is important to note the underlying assumptions. To this end, we acknowledge that (1) the stable isotope composition of fur keratin only correlates with the isotopic composition of food and water consumed during the moulting period of bats^52^. In migratory bats, including noctule bats, moulting occurs typically in summer, after the reproductive period of females and before migration between late July and early August^53,62^. Accordingly, the stable isotope composition of fur keratin is representative of the habitats where noctule bats live between late July and mid-August. If common noctule bats begin to migrate before moulting is completed, our stable isotope approach will provide less robust geographic assignments for the summer origin of bats^51^. (2) Our observation of common noctule bats feeding on insects with limnic larval stages suggests that δ^2^H values in fur keratin might be lower in these bats than in those with a diet of terrestrial insects^54^. Since our transfer function was obtained from bat species with a terrestrial diet, a diet of insects with limnic larval stages could potentially bias our geographic assignments to higher latitudes. A multi-species comparison of the isotopic composition of fur keratin in several aerial insectivorous bats showed that median δ^2^H values varied over a range of 20‰^54^, suggesting that we may have placed some of the long-distance migrants one or two isoclines further north than they originated (Fig. 4). Although we cannot refuse this possibility, we concur that our inferences are still robust, since a deviation by one or two isoclines still suggest a long-distance origin of bats from northeastern Europe, and since the cluster areas include several isocline zones (Fig. 3). (3) Our isoscape origin approach accounts for variation in stable isotope ratios in bat fur and also in precipitation water^63^. Therefore, assignment areas are relatively large. We acknowledge this low spatial resolution. However, given the migratory distances covered by some common noctules, our approach was sufficiently robust to separate long-distance migrants from regional bats. (4) Our sampling effort in France were concentrated along a west-east transect covering 500 km across France, and we obtained only a few carcasses in a given year. Therefore, we cannot infer the geographic origin and relative proportion of long-distance migrants in other regions of France, such as northern and southern France. Also, we cannot elaborate on intra- or inter-annual variation in the geographic origin of common noctules killed at wind turbines in France. We recommend that future studies should focus on this based on a larger sample size. Furthermore, (5) our conclusions are based on 60 individuals collected from four regions. Although we consider this sample size to be sufficient to draw robust conclusions, we acknowledge that the sample size varies along the west-east transect. We suggest larger sampling efforts at wind turbines across larger areas in Europe to generate a comprehensive picture on the migration of common noctule bats in Europe, and also about the potential impact wind energy production may have on distant populations of this migratory species. Lastly, (6) our study identifies the relative proportion of long-distance migrating noctule bats, but cannot quantify with empirical data the absolute number of long-distance migrating noctule bats killed by wind turbines in France.

Given these limitations, we estimate here the impact of wind energy production in France on migratory bats. To do this, we assume that mortality estimates from central Europe of 14 bats killed per wind turbine per year are representative for France^9^, which currently hosts about 9,700 onshore wind turbines with a total power capacity to 22.1 GW^64^. This leads to the inference that about 140,000 bats are killed per year at French wind turbines. In addition, we assume a relative proportion of about 18% for common noctule bats among bat carcasses found at wind turbines^65^, of which about 30% are long-distance migrants (this study^50^), . This corresponds to an estimated 25,200 common noctule bats killed at wind turbines per year in France, of which 17,640 are of regional and 7,560 of a north-eastern origin. This estimate should be refined on the basis of data from France, which is currently sparse or not easily accessible. For example, a report stated that only 6% of all bat carcasses at wind turbines in western France are attributable to common noctule bats^66^, and another report put the mortality rate of bats in France at only ~ 3–4 bats per wind turbine per year^67^. Furthermore, it has been claimed that about one third of wind turbines are operated with a curtailment scheme to protect bats^68^. Incorporating this data would lead to lower fatality estimates of migratory common noctule bats in France. Irrespective of this uncertainty, we argue that the total impact of bat fatalities at French wind turbines is greater than estimated for this single species, since the common noctule bat is only one of several vulnerable migratory bat species. Other migratory species such as Nathusius’ Pipistrelle (*Pipistrellus nathusii*), parti-coloured bats (*Vespertilio murinus*), Leisler’s Bat (*Nyctalus leisleri*), Greater Noctule (*Nyctalus lasiopterus*) and Schreiber’s Bat (*Miniopterus schreibersii*) have also been documented to be killed by wind turbines in France^65^. This highlights the urgent need for implementing mitigation schemes at the operating wind turbines in France as well as during the project development phase and environmental risk assessments of new onshore wind farms, by using established and highly effective curtailment protocols^27,69^. We plead for the implementation of these mitigation schemes in all European countries^9,43,44^.

## Conclusions

For our study sample of noctule bat carcasses collected from wind turbines in France, we show that about one third of these carcasses are migratory animals of distant geographical origin. The discovery of dead bats at wind turbines in France in general and the discovery of long-distance migrating bats in particular is conflicting with the legislation, both on the national (Loi de protection de la nature, § 76–629, 1976) and EU level (habitat directive, 92/43/EWG), and it also conflicts with international agreements, such as the CMS Convention (UNEP/EUROBATS agreement, signed Bonn 1979, and London 1991). We call for immediate action to implement efficient mitigation measures for newly installed wind turbines and also for retroactive implementation of mitigation measures for wind turbines already in operation^28^. These measures are important as the expansion scenarios for wind energy production in Europe envisage the installation of thousands of wind turbines in France and across Europe^9^. Failing to practice optimal siting of wind turbines, i.e. away from ecologically sensitive areas, and to implement curtailment schemes, may have far-reaching consequences for European biodiversity in general and vulnerable bat species in particular.

## Methods

### Study area and fur sample collection

**Fig. 5:**
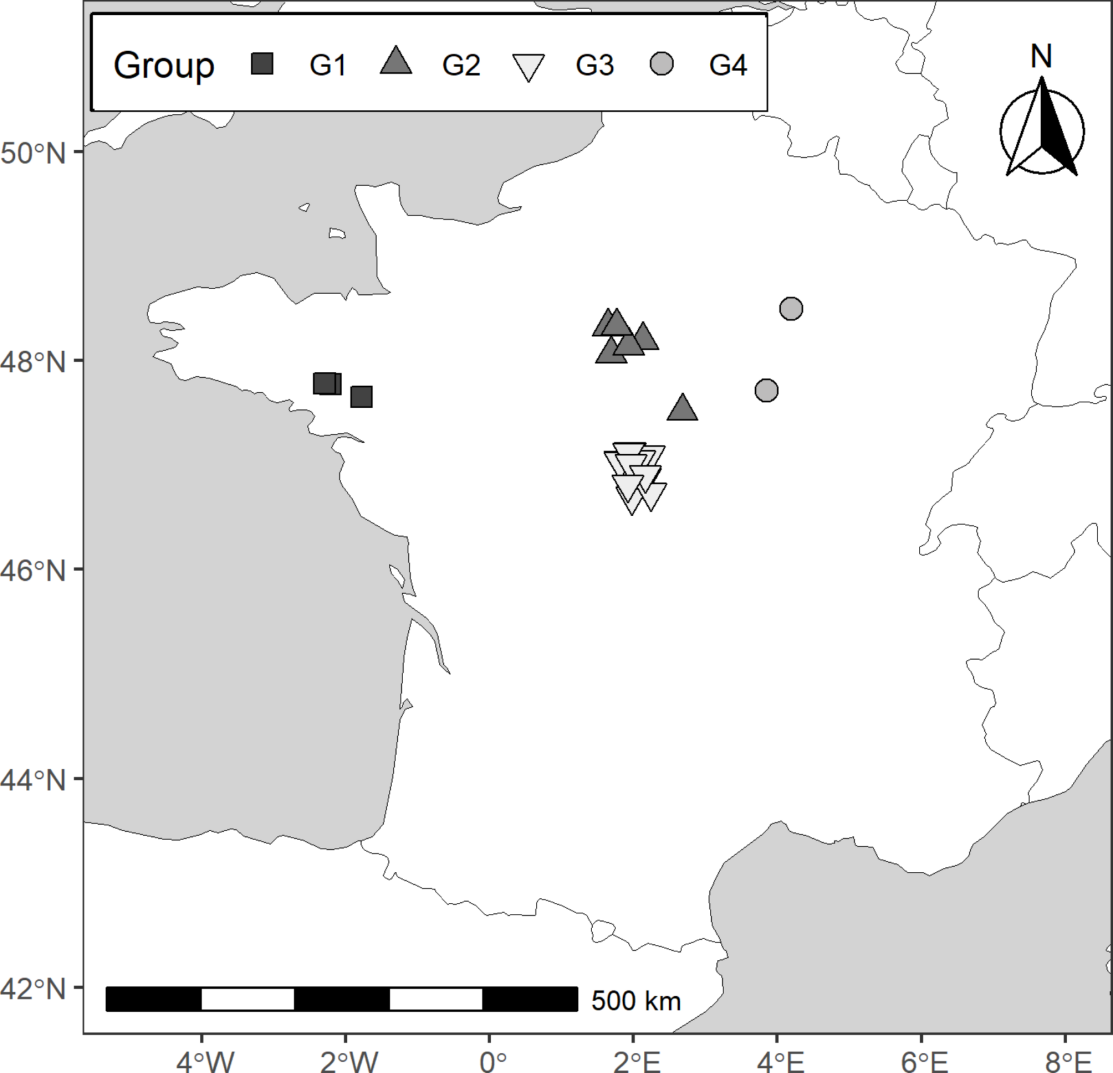
Geographic distribution of the sampled multi-wind turbine facilities and their respective groupings (G1 Bretagne/Pays-de-la-Loire: black squares; G2 and G3 Centre-Val de Loire (north and south): grey and white triangles, respectively; G4 Bourgogne-Franche-Comté/Grand-Est: grey circles).

We analysed fur samples collected from 60 common noctule bats (*Nyctalus noctula*) that were found dead under wind turbines during routine carcass searches following general monitoring schemes^20^. This monitoring was conducted by local environmental consulting firms and organizations between 2010 and 2023 at 23 multi-wind turbine facilities across three provinces in France, which includes the areas with the highest abundance of common noctules in France^70^: Bretagne/Pays-de-la-Loire (*n* = 3 surveyed multi-wind turbine facilities), Centre-Val de Loire (*n* = 18) and Bourgogne-Franche-Comté/Grand-Est (*n*= 2). All carcasses included in our study were collected between July and October, which covers the period when most bat carcasses are found at wind turbines in that region^66^. The collection sites were grouped into four geographic clusters based on location and proximity. Specifically, wind farms in Bretagne/Pays-de-la-Loire and Bourgogne-Franche-Comté/Grand-Est were classified in one group each, namely G1 and G4, respectively. Wind farms in Centre-Val de Loire were divided into G2 (central-north), and G3 (central-south), according to administrative boundaries (Fig. 5).

The fur samples were obtained by the Natural History Museum in Bourges (Centre-Val de Loire, France). From each carcass, we assessed the sex and age of the bat based on epiphyseal closure of finger bones where possible. Small tufts of fur were collected from the interscapular region using scissors and then transferred to dry Eppendorfs tubes for shipment to the stable isotope laboratory at the Leibniz Institute for Zoo and Wildlife Research (IZW, Berlin, Germany) for isotopic analysis. Juvenile bats were not included in our analysis due to potential age-related differences in isotopic composition between juvenile and adult bats^71^.

## Stable isotopes analysis

All fur samples were cleaned with a 2:1 chloroform-methanol solution to remove surface oils and contaminants, then dried in an oven at 50 °C for 24 h.

## Stable hydrogen isotope analysis

To account for the isotope exchange of hydrogen between fur keratin and ambient air, we used a comparative equilibration approach with the fur samples and previously calibrated keratin reference materials with ‘known’ non-exchangeable δ²H values^72^. For this purpose, we used in-house keratin laboratory reference materials, which included Swedish and Spanish sheep fur and Tanzanian goat fur (SWE-SHE; ESP-SHE; AFR-GOA, respectively). These in-house reference materials were previously calibrated against the revised and most updated isotope values of CBS, KHS, USGS42/43 ^73,74^.

Subsamples of bat fur and keratin reference materials were weighed with a microbalance to target a mass of about 0.3 mg of material, and placed in silver capsules (IVA Analysetechnik e.K., Meerbusch, Germany). These capsules were placed into microplates and left to equilibrate with lab ambient air for a week. After equilibration, samples were placed in an auto-sampler (Uniprep system^75^), flushed with helium, and combusted at 1350 °C in a high-temperature (HT) reactor of an elemental analyser (TCEA, Thermo Scientific; or HT pyrolysis system, HEKAtech). A gas chromatography separated the H_2_, N_2_, and CO gases produced during the pyrolysis. The resulting H_2_ from samples were measured for δ²H values using a continuous flow isotope ratio mass spectrometer (Delta V Advantage, Thermo Scientific, Bremen, Germany). Isotopic ratios were expressed in parts per mil (‰) deviations from the international standard: Vienna Standard Mean Ocean Water (VSMOW). The within-run analytical precision based on repeated analyses of keratin reference materials was better than 1.5‰ for δ²H.

### Stable carbon and nitrogen isotope analysis

For δ^13^C and δ^15^N analysis, fur samples were weighed to a target mass of 0.5 to 0.6 mg and placed in tin capsules (IVA Analysetechnik. Meerbusch, Germany). These subsamples were subsequently combusted at 1020 °C in an elemental analyser. The resulting N_2_ and CO_2 _gases were separated in a gas chromatography column and analysed with a continuous flow isotope ratio mass spectrometer (Delta V Advantage, Thermo Scientific, Bremen, Germany). Values of δ^13^C and δ^15^N were expressed in parts per mil (‰) deviations from international standards: Vienna Pee Dee Belemnite carbonate (V-PDB) for carbon and atmospheric nitrogen for nitrogen. The within–run analytical precision based on repeated analyses of keratin reference materials was better than 0.2‰ for δ^13^C, and 0.1‰ for δ^15^N values. We did not obtain meaningful data from one individual for δ^13^C and δ^15^N values and therefore the consecutive analysis was based only on 59 individuals.

### Statistical and spatial analysis

#### Transfer function

Using the R package ‘IsoriX’ (v. 0.9.2)^63^, we used a Linear Mixed Model to establish the transfer function between δ²H_p_ and δ²H in fur keratin (δ²H_f_) using the “*calibfit()*” function and a calibration dataset that included δ²H_f_values from sedentary bats of known origin (IsoriX: CalibDataBat)^51^. This calibration dataset contained δ²H_f_ values measured on 111 *Nyctalus noctula* during their sedentary period^55^, along with 224 bats from five non-migratory species (*Barbastella barbastellus*,* Eptesicus serotinus*,* Eptesicus isabellinus*,* Plecotus auritus*, and *Plectotus austriacus*)^59^. These published isotope values were re-calibrated to align with the current δ²H assigned values for keratin reference materials^73^, ensuring comparability with our isotope measurements. Using the European isoscape of predicted amount-weighted mean annual δ^2^H in precipitation and the stable hydrogen isotope values from 335 known-origin European bats^42,59^, we established the following transfer function for common noctule bats: δ^2^H_f_ = 11.91 (± 5.11) + 0.93 (± 0.09) * δ^2^H_p_.

By using the “*isoscape()*” function, we fitted a geostatistical mixed model including topographic features (elevation, longitude and latitude) to predict the variations in amount-weighted mean annual stable hydrogen isotope ratios in precipitation (δ²H_p_) across Europe. The δ²H_p_ values were obtained from the Global Network of Isotopes in Precipitation (GNIP) (*IAEA/WMO*,* 2024*) to build a European δ²H_p_ isoscape covering the distribution range of *Nyctalus noctula*. As our study is focused on a part of Europe (regional) and high altitude areas are not considered for this species’ origin, we expect that our model is a good approximation compared to other global improved models that include climatic variables^76^.

To separate bats of local origin from those of long-distance migratory origin, we employed the “*isofind()*” function in ‘*IsoriX’*. By applying the established δ²H_p_ isoscape and the transfer function between δ²H_p_ and δ²H_f_ values, we defined an isoscape of expected δ²H_f_ values for local bats across Europe (Fig. 4). Bats were classified as long-distance migratory if their δ²H_f_ values significantly differed from the expected δ²H_f_ values for local bats at their sampling location (P < 0.05). Conversely, if no differences was detected (P > 0.05), the local origin could not be ruled out, and the bats were considered as of regional origin.

### Variations in δ²H_f_, δ^13^C and δ^15^N values

All statistical analyses were performed with R (v. 4.3.2, R Core Team 2023). We used Shapiro-Wilk tests to assess the normality of the isotope values. We explored the relationship between the three stable isotope values (C, N, H) using Pearson’s correlations and Spearman rank correlation. T-tests and one-way ANOVAs (or Mann-Whitney U tests and Kruskal-Wallis tests, when appropriate) were performed to evaluate the effects of sex, sampling groups, and migratory behaviour (i.e., regional or long-distance migratory, see below) on the isotope values. To assess seasonal variations in bat fur isotope values including all years, the collection date was converted into “day of year” (Julian date).

### Geographic assignments based on δ²H_f_, values

We separated bats into clusters with similar δ²H_f _values using a k-means clustering analysis. The optimal number of clusters (k = 5) was determined when the within-cluster variation was only marginally reduced by the addition of a new cluster (elbow method)^77^. The probable geographic origin of bats within each cluster was inferred using group assignments in *IsoriX*^63^. The assignment probabilities of all individuals within the same cluster were combined for each location using Fisher’s method^78^, resulting in one map of probable origin per cluster. Using the same method, we produced a map of probable origin for all migratory individuals.

## Data Availability

The datasets used and/or analysed during the current study available from the corresponding author on reasonable request.
